# MALDI-TOF MS: optimization for future uses in entomological surveillance and identification of mosquitoes from New Caledonia

**DOI:** 10.1186/s13071-020-04234-8

**Published:** 2020-07-20

**Authors:** Antsa Rakotonirina, Morgane Pol, Malia Kainiu, Emilie Barsac, Jordan Tutagata, Sosiasi Kilama, Olivia O’Connor, Arnaud Tarantola, Julien Colot, Myrielle Dupont-Rouzeyrol, Vincent Richard, Nicolas Pocquet

**Affiliations:** 1 Institut Pasteur de Nouvelle-Calédonie, URE-Entomologie Médicale, Nouméa, 98845 New Caledonia; 2 Institut Pasteur de Nouvelle-Calédonie, Groupe de Recherche en Bactériologie Expérimentale, Nouméa, 98845 New Caledonia; 3 Institut Pasteur de Nouvelle-Calédonie, URE-Dengue et autres Arboviroses, Nouméa, 98845 New Caledonia; 4 Institut Pasteur de Nouvelle-Calédonie, URE-Epidémiologie, Nouméa, 98845 New Caledonia; 5 Institut Pasteur, Direction internationale, Paris, 75015 France

**Keywords:** Mosquitoes’ identification, MALDI-TOF mass spectrometry, New Caledonia

## Abstract

**Background:**

Mosquito vectors cause a significant human public health burden through the transmission of pathogens. Due to the expansion of international travel and trade, the dispersal of these mosquito vectors and the pathogens they carry is on the rise. Entomological surveillance is therefore required which relies on accurate mosquito species identification. This study aimed to optimize the use of matrix-assisted laser desorption/ionization time-of-flight mass spectrometry (MALDI-TOF MS) for mosquito identification.

**Methods:**

*Aedes aegypti* of the Bora-Bora strain and 11 field-sampled mosquito species were used in this study. Analyses were performed to study the impact of the trapping duration on mosquito identification with MALDI-TOF MS. The best preservation methods to use for short, medium and long-term preservation before MALDI-TOF MS analysis were also assessed. In addition, the number of specimens per species required for MALDI-TOF MS database creation was determined. The first MALDI-TOF database of New Caledonian mosquitoes was assembled and the optimal threshold for mosquito species identification according to the sensitivity and specificity of this technique was determined.

**Results:**

This study showed that the identification scores decreased as the trapping duration increased. High identification scores were obtained for mosquitoes preserved on silica gel and cotton at room temperature and those frozen at − 20 °C, even after two months of preservation. In addition, the results showed that the scores increased according to the number of main spectrum patterns (MSPs) used until they reached a plateau at 5 MSPs for *Ae. aegypti*. Mosquitoes (*n* = 67) belonging to 11 species were used to create the MALDI-TOF reference database. During blind test analysis, 96% of mosquitoes tested (*n* = 224) were correctly identified. Finally, based on MALDI-TOF MS sensitivity and specificity, the threshold value of 1.8 was retained for a secure identification score.

**Conclusions:**

MALDI-TOF MS allows accurate species identification with high sensitivity and specificity and is a promising tool in public health for mosquito vector surveillance.
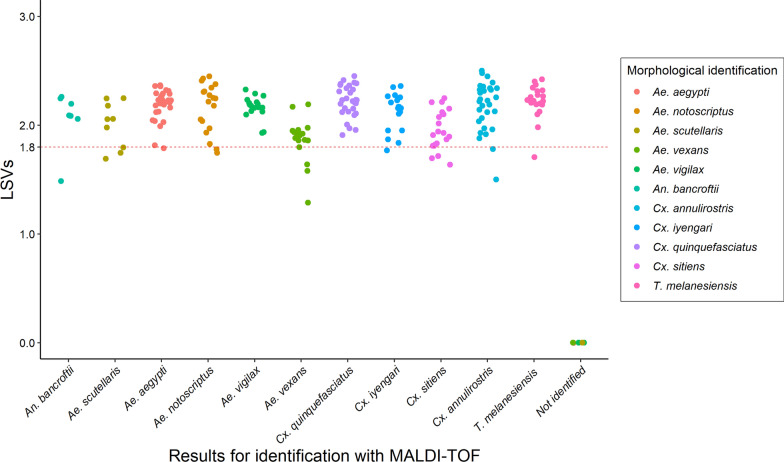

## Background

Vector-borne diseases are among the most significant public health burdens in the world. In addition to dengue fever which is responsible for 390 million infections per year, the emergence or re-emergence of yellow fever, chikungunya fever and Zika fever resulted in pandemics with significant morbidity [1–3]. These diseases are caused by arboviruses that are transmitted to humans through the bites of vector mosquitoes.

New Caledonia is a subtropical island located in the southwestern Pacific Ocean where dengue is the most prevalent arthropod-borne viral infection with epidemics occurring regularly [4]. Chikungunya and Zika infections have been also reported on the territory [2, 5]. The major vector of these arboviruses is *Aedes aegypti.* There is also a risk of other arboviruses being introduced in New Caledonia such as West Nile virus, Rift Valley fever virus, Japanese encephalitis virus or Ross River virus, as suitable vector species are present: *Culex quinquefasciatus*; *Culex annulirostris*; *Culex sitiens*; *Aedes vigilax*; *Aedes vexans*; and *Aedes notoscriptus* [6–10]. In addition, the risk for introduction of exotic mosquito species is also important because other vector species are documented in neighboring countries [11–13]. For example, two vector species were recently introduced: *Aedes scutellaris*, a vector of dengue virus, detected in 2016 and *Anopheles bancroftii*, a secondary vector of *Plasmodium* sp., detected in 2017 [14, 15].

Given the threat of introduction of both exotic vector mosquito species and other arboviruses in New Caledonia, enhanced surveillance is crucial. Strengthening of surveillance has been identified by the World Health Organization as one of the four pillars of effective vector control in the Global Vector Control Response plan for 2017–2030 [16]. This surveillance relies on accurate mosquito species identification. Indeed, knowledge of existing vector mosquito species on the territory allows a better risk assessment for vector borne diseases. Rapid detection thanks to reliable identification of introduced vector species limits their installation through timely and adequate vector control measures.

Several techniques can be used to identify mosquitoes. Morphological identification is widely used, it is inexpensive and can be readily conducted in the field [17]. However, it requires expertise in entomology that is often unavailable. Furthermore, morphological identification is difficult due to the similarity within cryptic species and/or damage to the specimen during sampling [17, 18]. The molecular method, specifically DNA sequencing, is necessary when mosquitoes belong to cryptic species complexes that cannot be distinguished morphologically. This method is one of the most sensitive and specific techniques but requires pre-existing DNA sequence information for species identification [17]. This method remains also expensive and time consuming; it is therefore not suitable for routine mosquito identification [19].

Recently, matrix-assisted laser desorption ionization time-of-flight mass spectrometry (MALDI-TOF MS) was introduced for mosquito species identification [19, 20]. This method generates unique protein mass spectra, “fingerprints” specific to each species [21]. When the spectrum is compared to a database of reference spectra, the software calculates a score that indicates the reliability of the identification provided. The score is then compared to a threshold value and allows species identification [21]. This method is much less costly than the molecular method [22]. In addition, the MALDI-TOF MS technique can be used for mosquito species complex identification (i.e. morphologically indistinguishable species) [19, 20].

Although this technique is promising for mosquitoes’ identification, some parameters have to be considered prior to MALDI-TOF MS analysis. Indeed, previous studies on several arthropods have underlined the impact of trap type used for sampling, the preservation methods and the body part used for protein extraction on the spectra profile [23–26]. The impact of trapping duration on MALDI-TOF identification, however, has never been evaluated. In addition, no clear consensus for the minimum number of specimens per species required for a robust MALDI-TOF database is available. Regarding the threshold value for mosquito species identification, there is no value registered by the software manufacturer. The threshold value at 1.8 established following mosquito blind testing [19] should be confirmed to allow routine mosquito species identification with MALDI-TOF MS.

The overall aim of this study was to optimize the use of MALDI-TOF MS for mosquito identification, while taking into account the parameters previously described. The specific objectives were: (i) to determine whether trapping duration affects MALDI-TOF MS identification; (ii) to determine the best methods to use for short, medium and long-term preservation before MALDI-TOF MS analysis; and (iii) to determine the number of specimens per species required for the MALDI-TOF MS database creation. The threshold value for mosquito identification with the database was also determined, based on MALDI-TOF MS sensitivity and specificity.

## Methods

### Biological material

Laboratory-reared mosquitoes were used to measure the effect of trapping duration and preservation methods on MALDI-TOF MS results. *Aedes aegypti* (Bora-Bora strain) were raised in the insectarium at 28 °C ± 2 °C and a relative humidity of 80 ± 10%. Larvae were reared until the pupal stage in a plastic tray containing 1.5 l of water and fed with beer yeast *ad libitum*. Pupae were then collected and transferred to a mosquito cage. Adults that emerged were fed with a 10% glucose solution and were harvested when they were 3–5-days-old and killed before MALDI-TOF MS analysis.

Eleven species of field-collected mosquitoes were used to create and validate the MALDI-TOF database (Table 1). Field-collection of both larval and adult stages was undertaken from March 2016 to April 2019. Different sampling methods were used to collect adults according to the target species: CDC light trap with CO_2_; BG-sentinel trap (Biogents); and human landing catch. During these samplings, traps were set for a maximum of 24 h. Larvae were collected in their breeding sites and raised to adults in the laboratory. All these mosquitoes were preserved at − 20 °C, or at − 80 °C if storage exceeded 3 weeks before MALDI-TOF MS analysis.

**Table 1:** Mosquitoes used for MALDI-TOF MS analysis

Species	No. of specimens in database	No. of specimens used in blind testing	Species identified *via* GenBank	*cox*1 [31] sequence similarity (%)	ITS2 [32] sequence similarity (%)
*An. bancroftii*	3	7	*An. bancroftii*	na	100
*Ae. scutellaris*	5	10	*Ae. scutellaris*	> 99	na
*Ae. aegypti*	10	30	*Ae. aegypti*	> 99	na
*Ae. notoscriptus*	5	19	*Ae. notoscriptus*	> 96	na
*Ae. vigilax*	5	20	*Ae. vigilax*	> 99	na
*Ae. vexans*	5	20	*Ae. vexans*	> 99	na
*Cx. quinquefasciatus*	9	30	*Cx. quinquefasciatus*	> 98	na
*Cx. iyengari*	5	18	na	na	na
*Cx. sitiens*	5	20	*Cx. sitiens*	> 99	na
*Cx. annulirostris*	10	30	*Cx. annulirostris*	> 97	na
*T. melanesiensis*	5	20	na	na	na
Total	67	224	–	–	–

*Abbreviation:* na, not available

### Morphological and molecular identification of mosquitoes

All mosquitoes used in this study were morphologically identified using dichotomous identification keys before all experiments [11, 27–29]. As no mosquito species complex is currently reported to be present in New Caledonia, all mosquito species were morphologically distinguishable. Morphological identification was confirmed through double-blind evaluation by experienced entomologists.

In addition to morphological identification, molecular analysis was also performed for all the specimens used as references for the creation of the MALDI-TOF database. For this, the head, thorax and abdomen of each mosquito were removed and transferred in 1.5 ml microtubes for DNA extraction. DNA was extracted using a DNA Blood and Tissue Kit (Qiagen, Hilden, Germany) according to the manufacturer’s recommendation.

The extracted DNA was subsequently amplified by polymerase chain reaction (PCR) using specific primers targeting the cytochrome *c* oxidase subunit 1 (*cox*1) region: LCO1490 (forward: 5’-GGT CAA CAA ATC ATA AAG ATA TTG G-3’) and HC02198 (reverse: 5’-TAA ACT TCA GGG TGA CCA AAA AAT CA-3’) [30]. Primers targeting the internal transcribed spacer 2 (ITS2) region of the ribosomal RNA gene cluster (forward: 5’-TGT GAA CTG CAG GAC ACA T-3’; reverse: 5’-TAT GCT TAA ATT CAG GGG GT-3’) were also used for the molecular identification of *An. bancroftii* [31]. The PCR conditions were: 0.2 µM forward primer; 0.2 µM reverse primer; 1× master mix (Qiagen); distilled water; and template DNA (between 10 ng/µl and 100 ng/µl). The PCR was performed in a total volume of 25 µl under different thermal conditions according to the primers used. To amplify *cox*1, cycling involved an initial denaturation at 94 °C for 3 min, 35 cycles of 94 °C for 1 min, 50 °C for 1 min and 72 °C for 1 min, followed by a final extension step at 72 °C for 10 min. To amplify ITS2, the thermal conditions were: 4 min at 94 °C, 35 cycles of 1 min at 94 °C, 120 s at 51 °C and 1 min at 72 °C, and a final extension step at 72 °C for 10 min.

Finally, PCR products were sequenced (Genoscreen, Lille, France) and the sequences were assembled and analyzed using PREGAP and GAP software (version 4.10.2, 2019; The GAP Group, GAP-Groups, Algorithms and Programming, Aachen, Germany). Obtained sequences were compared with mosquito sequences available in the GenBank database using the BLAST platform (http://blast.ncbi.nlm.nih.gov/Blast.cgi).

### Protein extraction for MALDI-TOF analysis

For protein extraction, the experiment was focused using only legs, which is the widely used method for MALDI-TOF MS mosquito database creation. This reduces bias caused by potential traces of blood-meals or microbiota in the spectra [19]. The sample preparation method used was adapted to the protocol described by Raharimalala et al. [32]. All the legs of each mosquito were removed and rinsed in a microtube with 1 ml of 70% ethanol for 60 s, followed by 1 ml of distilled water for 60 s. After total elimination of water, 3 metal beads of 2.4 mm diameter, 15 µl of acetonitrile (50%) and 15 µl of formic acid (70%) were added to the microtube. Each sample was subsequently homogenized using a MagnaLyser, version 1.1 (Roche, Mannheim, Germany) with 3 cycles of 30 s at a frequency of 3000× *rpm*. The homogenates were transferred into 1.5 ml Eppendorf microtubes and centrifuged at 10,000× *g* for 2 min. To improve deposit homogeneity and ensure the rapidity of spectra acquisition, 1 µl of sample was deposited directly on a steel MALDI plate (Bruker Daltonics, Wissembourg, France) and allowed to dry before adding another 1 µl of the same sample over the first spot. As per a previous study on mosquito identification with MALDI-TOF, 8 spots of the same sample were deposited on the plate to create the reference main spectrum patterns (MSP) [32]. Conversely, only one spot was deposited for each sample intended to be queried against these reference spectra in order to assess all MALDI-TOF analyses without score optimization caused by multiple spots. After drying at room temperature, all spots were recovered with 1 µl of matrix solution (α-cyano-4-hydroxycynamique solubilized into 50% acetonitrile, 2.5% trifluoroacetic and 47.5% high-performance liquid chromatography-grade water; Honeywell, North Carolina, USA). Matrix solution, deposited in duplicate on the plate, was used as a control for matrix quality. Finally, the plate was dried at room temperature before MALDI-TOF MS analysis.

### MALDI-TOF parameters and analysis

Spectra ranging from 2000 to 20,000 Daltons were acquired using a Microflex MALDI-TOF mass spectrometer (Bruker). Measurements were performed with flexControl software, version 3.3 (Bruker) with detection in the linear positive-ion mode at a laser frequency of 60 Hz and laser power between 40–50%. Each spectrum obtained corresponds to an accumulation of 240 laser shots from the same spot performed in 6 regions.

For each reference sample used to create MSP, the 8 replicates were individually measured 3 times. The quality of the spectra obtained was verified with MALDI flex analysis software, version 3.3 (Bruker). Then, a selection of 20–24 high quality mass spectra per sample, obtained with the 8 spots measured 3 times, were imported into the MALDI Biotyper compass software, version 3.1 (Bruker). MSP containing the average of peak mass, the average of peak intensity and peak frequency information was subsequently calculated for each reference sample.

For the sample intended to be queried against the MSP, one measurement was realized for each spot (one spot per sample). The mass spectrum of each sample was compared to the MSPs in the MALDI-TOF database with the MALDI Biotyper compass software. This software calculates a log-score value (LSV) ranging from zero to three, reflecting the similarity between sample spectra and MSP. A Log-score value greater than or equal to a threshold value supports the accuracy of the sample identification.

### Effect of trapping duration on MALDI-TOF identification

To assess only the impact of trapping duration on mosquito identification with MALDI-TOF MS and to limit inter-individual variability, *Ae. aegypti* females belonging to the same laboratory strain (Bora-Bora strain) and the same generation were used. These were raised in the same laboratory conditions. Five BG-sentinel traps were placed in field conditions. Mosquitoes were quickly anesthetized at 4 °C and 30 alive specimens were placed in each trap. Then, each trap’s funnel was covered up to avoid the introduction of other mosquitoes. Traps were harvested after 24, 48, 72, 96 and 168 h, respectively. Fresh specimens (*n* = 30) were also included in the analysis. Each mosquito was subjected to MALDI-TOF MS analysis. To limit the impact of the multiple MSPs on MALDI-TOF results, they were compared with only one MSP created from a fresh specimen.

### Determination of the best methods of preservation

Three preservation methods were studied: (i) freezing at − 20 °C; (ii) preservation in 70% ethanol; and (iii) preservation on silica gel and cotton at room temperature (*c.*25 °C). A total of 338 *Ae. aegypti* females belonging to the same laboratory strain (Bora-Bora strain), the same generation and raised in the same laboratory condition were also included in analysis. Mosquitoes were sampled and individualized in 1.5 µl microtubes. They were preserved using each of the 3 preservation methods described, for 9, 32, 62 and 218 days. Thirty fresh specimens were also included in the analysis. All these mosquitoes were submitted individually to MALDI-TOF MS analysis, and compared with one MSP created from a fresh specimen to limit score optimization due to multiple MSPs being included.

### Determination of the required number of MSPs per species for database creation

Analysis was performed in order to determine the required number of specimens per species for MALDI-TOF MS database creation. The assessment was investigated for three species from the field: *Ae. aegypti*; *Cx. quinquefasciatus*; and *Cx. annulirostris*. Nine MSPs were created for *Cx. quinquefasciatus* while 10 MSPs per species were created for *Ae. aegypti* and *Cx. annulirostris*. The spectra of 30 mosquitoes per species were compared with 1 to 9 corresponding MSPs for *Cx. quinquefasciatus* and from 1 to 10 MSPs for the other species. The objective was to test the number of required MSPs to obtain the best identification score. The LSV median was computed for the 3 species for each number of MSPs tested.

### Creation and validation of the MALDI-TOF MS database of mosquitoes from New Caledonia

The findings observed during these previous steps were taken into consideration during the database creation. For this, 11 mosquito species collected in the field were used to create the MALDI-TOF database of New Caledonian mosquitoes. A total of 67 fresh specimens were used to create MSPs entered in the database.

Seven to 30 specimens from the field belonging to these 11 species underwent MALDI-TOF analysis to evaluate the accuracy of this newly created MALDI-TOF database. Their spectra were blindly queried, regardless of the presence of the species’ MSPs in the database. The reliability of species identification was estimated using the LSVs obtained from the MALDI Biotyper software (Bruker).

### Determination of the threshold value for mosquito identification with MALDI-TOF

The database was used as a reference to determine the threshold value for which both the sensitivity and the specificity of the MALDI-TOF identifications were optimal. This was achieved by comparing the spectra of field-collected specimens in a blinded experiment. In this process, all the field specimens belonging to the 11 species were first blindly queried against MSPs for each species individually. Then, these were also queried against MSPs for a random selection of 6 species on the one hand, and of the 5 other species on the other hand. For these analyses, sensitivity (Se) and specificity (Sp) were calculated at various threshold values, ranging from 0 to 3. In addition, positive and negative predictive values were determined.

The receiver operating characteristics (ROC) curve was also established. For this, area under the curve (AUC) values were evaluated to assess the discrimination of the MALDI-TOF MS analysis compared with the morphological technique.

### Statistical analysis

Statistical analysis was performed using R software (R Core Team (2017). R: A language and environment for statistical computing. R Foundation for Statistical Computing, Vienna, Austria). A non-parametric Wilcoxon test was carried out to compare LSV medians. A Kruskal-Wallis non-parametric test was also used to compare medians across multiple groups. The statistical significance threshold for these tests was set at 0.05. The ROC analysis and graphs were also developed with R software.

## Results

### Mosquito identification

The mosquitoes used in this study were morphologically identified as belonging to 11 different species: *An. bancroftii* (*n* = 10); *Ae. scutellaris* (*n* = 15); *Ae. aegypti* (*n* = 40); *Ae. notoscriptus* (*n* = 24); *Ae. vigilax* (*n* = 25); *Ae. vexans* (*n* = 25); *Cx. quinquefasciatus* (*n* = 39); *Cx. iyengari* (*n* = 23); *Cx. sitiens* (*n* = 25); *Cx. annulirostris* (*n* = 40) and *Tripteroides melanesiensis* (*n* = 25).

*cox*1 sequences were generated for all the 67 mosquitoes used to create the reference database. *Anopheles bancroftii* ITS2 spacer was also sequenced. The *cox*1 query and ITS2 sequences in the GenBank database allowed us to obtain reliable species identification for all the mosquito species of which reference sequences were available (Table 1); no *cox*1 sequences were available on GenBank for *An. bancroftii*, *Cx. iyengari* and *T. melanesiensis*. All sequences obtained in this study are available in GenBank database under the accession numbers MN733743-MN733814.

### Impact of trapping duration on MALDI-TOF MS results

**Fig. 1:**
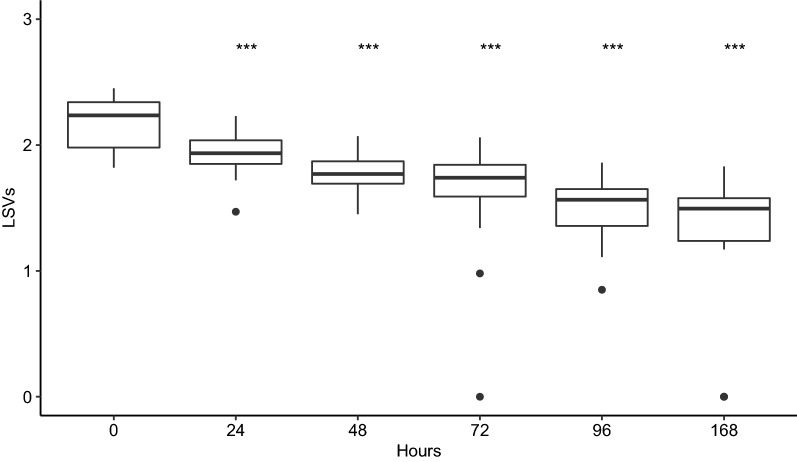
Impact of trapping durations on *Ae. aegypti* identification scores. Comparison of log-score values (LSVs) of fresh mosquitoes (*n* = 30) and mosquitoes trapped from 24 h to one week (*n* = 30 per point). Wilcoxon test, ***P < 0.001

MALDI-TOF MS spectra for protein extracts from fresh mosquitoes (*n* = 30) and specimens trapped inside of BG-sentinel for durations ranging from 24 h to 1 week (30 per trapping duration) were compared with one fresh mosquito MSP. High LSVs were obtained for the fresh specimens (median of 2.23). The LSVs decreased as trapping duration increased (Fig. 1). Despite the significant difference between the mosquitoes trapped during 24 h and fresh specimens (Wilcoxon test: *Z* = 4.28, *P* = 1.92 × 10^−5^), LSV medians remained above 1.8 (i.e. 1.93). The comparison between spectra for fresh mosquitoes and for those harvested at each time point showed modification of spectra profile even after 24 h of trapping (Additional file 1: Figure S1).

### Effect of mosquitoes’ preservation method and duration on MALDI-TOF results

**Fig. 2:**
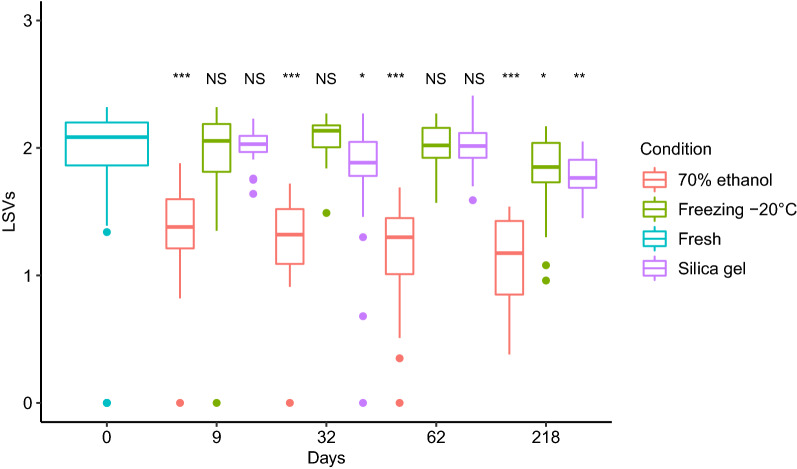
Impact of methods and durations of preservation on *Ae. aegypti* identification scores. Comparison of LSVs of fresh mosquitoes (*n* = 30) and mosquitoes preserved in 70% ethanol, at *− *20 °C and on silica gel and cotton at room temperature from 9 to 218 days (*n* = 18 to 30 per duration and per condition). Wilcoxon test, *P < 0.05, **P < 0.01, ***P < 0.001. *Abbreviation*: NS, not significant

For each preservation method, 18–30 *Ae. aegypti* per day point were submitted individually to MALDI-TOF MS. Thirty fresh mosquitoes were also included in this experiment. Their spectra were compared to one MSP created from a fresh reference sample. The results showed a high LSV median (2.08) for the fresh mosquitoes (Fig. 2). The same result was observed for mosquitoes preserved on silica gel and cotton and those frozen during 62 days: LSV medians were of 2.01 and 2.02, respectively. LSV medians for these mosquitoes did not differ significantly from those for fresh mosquitoes (Kruskal-Wallis H-test: *χ*^2^**=** 0.3, *df* = 2, *P* = 0.8). Despite the significant difference between the mosquitoes preserved on silica gel and cotton during 32 days and fresh specimens (Wilcoxon test: *Z* = 2.06, *P* = 0.04), LSV medians remained above 1.8 (i.e. 1.89). At 218 days of preservation, the LSV medians decreased for frozen mosquitoes and those preserved on silica gel and cotton. However, the LSV medians in frozen specimens remained greater than 1.8 (i.e. 1.85) despite their significant difference with fresh mosquitoes (Wilcoxon test: *Z* = − 2.29, *P* = 0.02). Low LSVs were observed for mosquitoes preserved in 70% ethanol, including at day 9 for which the LSV median was 1.38. These differed significantly from those found in fresh mosquitoes (Wilcoxon test: *Z* = − 4.31, *P* = 1.6 × 10^−5^).

### Number of required MSPs per species to obtain the best identification score

**Fig. 3:**
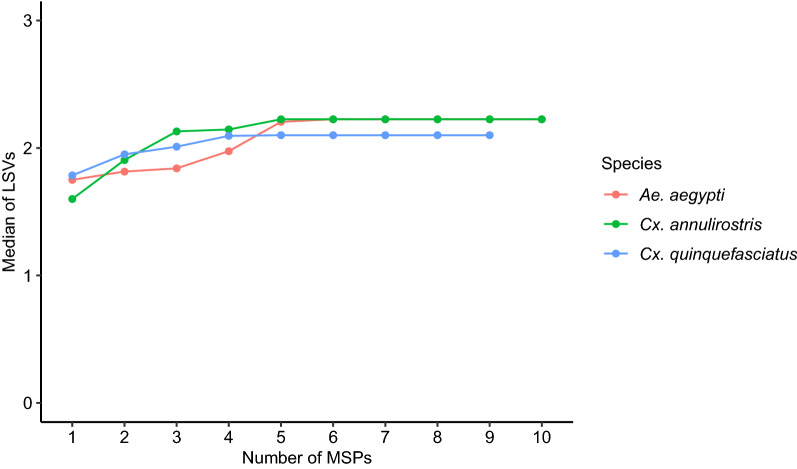
Progression of log-score values according to the number of MSPs used in the analysis. Three mosquito species from the field were used, *Ae. aegypti*, *Cx. annulirostris* and *Cx. quinquefasciatus*. X-axis corresponds to the number of MSPs used in the database. Y-axis corresponds to the median of LSVs. Colors illustrate each species tested

Before the creation of the MALDI-TOF database, analysis was performed to determine the number of MSPs required per species for the optimization of LSVs. Three species were used for the experiment: *Ae. aegypti*; *Cx. annulirostris*; and *Cx. quinquefasciatus*. For all three species, the analysis showed that the LSV medians increased according to the number of MSPs used until they reached a plateau (Fig. 3). For *Ae. aegypti*, this plateau was reached after using 5 MSPs. Indeed, no significant difference was observed between the LSV medians when using 5–10 MSPs (Kruskal-Wallis test H-test: *χ*^2^**=** 0.7, *df* = 5, *P* = 1). The plateau was reached when using 3 MSPs for *Cx. annulirostris* and *Cx. quinquefasciatus*: no significant difference was observed when using 3–10 MSPs (Kruskal-Wallis test H-test: *χ*^2^**=** 3.8, *df* = 7, *P* = 0.8 for *Cx. annulirostris* and Kruskal-Wallis test H-test: *χ*^2^**=** 10.3, *df* = 6, *P* = 0.1 for *Cx. quinquefasciatus*).

### Creation and validation of MALDI-TOF MS database

Five MS reference spectra per species were used to create the database except for four species. Only 3 *An. bancroftii* MSPs were created due to the small number of specimens available (Table 1). Further MSPs were created for *Ae. aegypti* (*n* = 10), *Cx. quinquefasciatus* (*n* = 9) and *Cx. annulirostris* (*n* = 10). These were created during the previous step (i.e. determination of the number required of MSPs per species) and were not removed from the database during analysis. The visualization of the relationship between MSPs in the database displayed in a dendrogram showed that MSPs of each species clustered together, with distance levels under 500 (Additional file 2: Figure S2). A total of 224 field-collected mosquitoes belonging to 11 species, consisting of 7–30 specimens per species, were queried against this database. All these mosquitoes used to create and validate the MALDI-TOF database were analyzed no later than 5 months after sampling, except for *Ae. scutellaris* and *An. bancroftii* which were collected in 2016 and 2017, respectively (Table 1) and were preserved at − 80 °C.

**Fig. 4:**
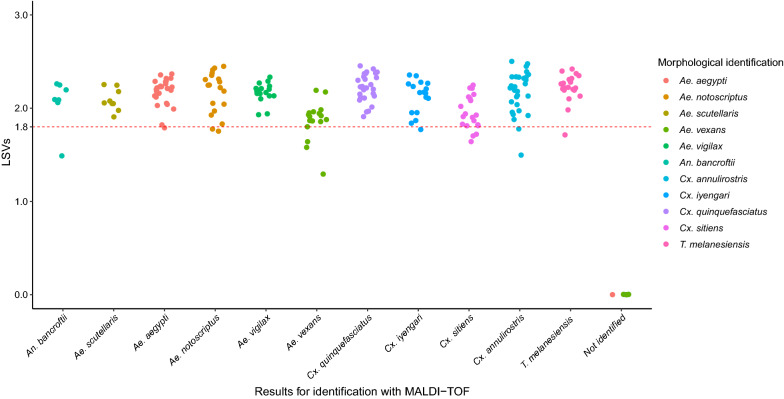
Comparison of the results obtained with morphological identification and MALDI-TOF MS analysis. X-axis corresponds to the results obtained with MALDI-TOF analysis. Y-axis corresponds to LSVs. Colors illustrate morphological identification obtained through double-blind evaluation

Except for the 9 mosquitoes for which no spectrum was obtained, all the specimens were matched with MSP of the correct species in the database. Specifically, reliable matching was acquired for 96% (95% CI: 93–98%) of the specimens from each species during the blind test analysis (Fig. 4). Species in the same group matched also correctly including 100% of the *Sitiens* group (*Cx. sitiens* and *Cx. annulirotris*) and 100% of the *Pipiens* group (*Cx. quinquefasciatus* and *Cx. iyengari*).

Other analyses were performed to determine the identification threshold value for these mosquito species and calculated MALDI-TOF MS sensitivity and specificity values. The results showed that the optimal threshold value which maximized the MALDI-TOF MS sensitivity and the specificity ranged from 1.48 to 1.79 when only one species was used in the database (Table 2). When approximately half the species in the database were included in the analysis, this threshold value ranged from 1.63 to 1.7 (Table 2; Additional file 3: Figure S3). High positive predictive values and negative predictive values were obtained (Table 2). High AUC (area under the curve) values (0.85 to 1) were found regardless of the species used.

**Table 2:** Overview of results obtained during the determination of the optimal threshold for MALDI-TOF MS identification

Species in the database	Optimal threshold	TP	FN	TN	FP	Sensitivity	Specificity	PPV	NPV	AUC	95% CI (AUC)
*An. bancroftii*	1.48	7	0	217	0	1	1	1	1	1	1–1
*Ae. scutellaris*	1.75	9	1	214	0	0.9	1	0.9	1	0.90	0.77–1
*Ae. aegypti*	1.77	18	1	205	0	0.97	1	0.95	1	0.97	0.92–1
*Ae. notoscriptus*	1.75	19	0	205	0	1	1	1	1	1	1–1
*Ae. vigilax*	1.57	17	3	204	0	0.85	1	0.85	1	0.85	0.75–0.96
*Ae. vexans*	1.57	16	4	204	0	0.8	1	0.80	1	0.85	0.75–0.96
*Cx. quinquefasciatus*	1.79	29	1	190	4	0.97	0.98	0.97	0.98	0.97	0.92–1
*Cx. iyengari*	1.77	18	0	205	1	1	0.99	1	0.99	0.99	0.99–1
*Cx. sitiens*	1.64	20	0	214	0	1	1	1	1	1	1–1
*Cx. annulirostris*	1.78	29	1	190	4	0.97	0.98	0.97	0.98	0.99	0.97–1
*T. melanesiensis*	1.7	20	0	204	0	1	1	1	1	1	1–1
Six species^a^	1.70	109	8	100	7	0.93	0.93	0.93	0.93	0.94	0.91–0.97
Five species^b^	1.63	101	6	110	7	0.94	0.95	0.94	0.94	0.96	0.94–0.99

^a^*An. bancroftii, Ae. scutellaris*, *Ae. aegypti*, *Ae. vigilax*, *Cx. quinquefasciatus*, *Cx. sitiens*

^b^*Ae. notoscriptus*, *Ae. vexans*, *Cx. iyengari*, *Cx. annulirostris*, *T. melanesiensis*

*Abbreviations*: AUC, area under the curve; TP, true positives; FN, false negatives; TN, true negatives; FP, false positives; PPV, positive predictive values; NPV, negative predictive values

## Discussion

MALDI-TOF MS is an innovative tool for arthropod identification [17]. To optimize its use for routine mosquito identification and entomological surveillance, some parameters must be taken into consideration. In the present study, the effect of trapping duration and three preservation methods on MALDI-TOF results were evaluated. To the best of our knowledge, the required number of specimens per species for MALDI-TOF MS database creation and the optimal threshold for mosquitoes’ identification were determined for the first time. The first MALDI-TOF MS reference spectra for the identification of a selection of New Caledonian mosquito species is presented in this study.

In entomological surveillance, traps are sometimes deployed for a long period in the field. Mosquitoes therefore remain trapped several days and exposed to uncontrolled temperature and relative humidity variations. Here, the consequence of this trapping duration on MALDI-TOF MS analysis was assessed. The results show that long-term trapping could alter the identification score for MALDI-TOF MS identification. This alteration was important from 48 hours of trapping. Indeed, at 24 hours of trapping, the LSV medians remained above the threshold value for mosquito identification (i.e. 1.8) and 87% of the mosquitoes had LSVs greater than or equal to this threshold. This alteration of MALDI-TOF results may be explained by the mosquitoes’ exposure to variations in relative humidity in the BG-trap. According to these findings, the trapping duration should therefore not exceed 24 hours for reliable identification using MALDI-TOF MS.

The mosquitoes collected from field traps may sometimes be preserved for several weeks or months before MALDI-TOF MS identification. The results show that preserving mosquitoes in ethanol alters the MALDI-TOF MS identification results: low LSVs for the mosquitoes preserved in 70% ethanol was found, even after preservation for nine days. These observations are certainly related to the decrease of protein solubility which could lead to the qualitative and quantitative loss in spectra profiles, as others have shown [33]. To circumvent these limitations, some authors have suggested the query of specimens against MS reference spectra of specimens similarly preserved in ethanol for the same duration [25, 34, 35]. In supplementary experiments, LSVs improvement of specimens preserved in 70% ethanol was observed, when they were compared with MSP created from mosquitoes preserved under similar conditions for one day (Additional file 4: Figure S4a). In all cases, however, fast LSVs degradation was observed for the ethanol group. In addition, the LSVs for mosquitoes of this group remained lower than those of frozen specimens (Additional file 4: Figure S4b) and mosquitoes preserved on silica gel and cotton (Additional file 4: Figure S4c).

The second preservation method evaluated was the preservation on silica gel and cotton. Stable LSVs for the mosquitoes preserved with this method was observed, even after two months of preservation. This LSV stability could be linked to the property of silica gel in controlling the relative humidity of the samples [36]. This preservation method is therefore suitable for mosquito species identification with MALDI-TOF MS. In addition, this method is operationally and financially more accessible than the freezing method for transporting samples from the field to the laboratory. Other work showed that this preservation method is also suitable for identification of mosquito blood-meal sources [26].

The third preservation method tested in this study was − 20 °C freezing. The results showed high LSVs for the frozen mosquitoes even after 62 days of preservation. In agreement with previous works, these findings suggest that freezing at − 20 °C is one of the best preservation methods [17, 25]. A decrease of LSVs was however observed after seven months of freezing (day 218), with only 60% of the mosquitoes having LSVs greater than or equal to the 1.8 threshold. During an additional experiment, the spectra obtained from *Ae. aegypti* preserved at − 80 °C during four years were also compared to MSPs of fresh field-collected mosquitoes (Additional file 5: Figure S5). All these mosquitoes (*n* = 20) were correctly identified with LSVs greater than 1.8. Storage at − 80 °C is therefore compatible as a long-term preservation method before MALDI-TOF MS analysis.

To the best of our knowledge, the required number of specimens per species needed to create a MALDI-TOF MS mosquito database was determined for the first time. The assessment for three field-collected species showed that optimal LSVs were achieved when using three MSPs for *Cx. quinquefasciatus* and *Cx. annulirostris* and five MSPs for *Ae. aegypti* (Fig. 3); for optimal LSVs, at least five MSPs per species were used to create this database except for *An. bancroftii* because of the low number of available individuals.

This MALDI-TOF database included only field mosquitoes identified into 11 species. Two of these mosquito species were recently introduced in New Caledonia: *Ae. scutellaris*, vector of dengue virus; and *An. bancroftii*, vector of *Plasmodium* sp. [14, 15]. Other species included in the database are also vectors: *Ae. aegypti* is the main vector of dengue, Zika and chikungunya viruses in the Pacific region [37]; *Cx. quinquefasciatus*, *Cx. annulirostris*, *Cx. sitiens*, *Ae. notoscriptus*, *Ae. vigilax* and *Ae. vexans* are known vectors of other viruses such as West Nile virus, Rift Valley fever virus, Japanese encephalitis virus and Ross River virus [6–10]. In addition, some of these species are morphologically similar to one another: *Cx. quinquefasciatus/Cx. iyengari* (*Pipiens* group); and *Cx. sitiens/Cx. annulirostris* (*Sitiens* group).

Using a cut-off distance level of 500 set by previous studies, all MSPs species in this database were reliably classified [38]. During blind testing, with the exception of the nine specimens for which no spectrum was obtained, all mosquitoes were correctly identified to the species level using MALDI-TOF MS. The absence of spectrum for these nine specimens is likely to be attributable to a few legs being available (i.e. less than four legs) which could lead to a failure to acquire sufficient protein for analysis. No mismatch was observed even for the species belonging to the same group such as *Cx. quinquefasciatus* and *Cx. iyengari* (*Pipiens* group) or *Cx. sitiens* and *Cx. annulirotris* (*Sitiens* group). Likewise, differentiation of mosquitoes within a species complex was also achieved using MALDI-TOF MS [19]. These observations show that MALDI-TOF MS is an effective method to identify cryptic mosquito species. MALDI-TOF MS could also distinguish species which are morphologically closely related but with a dissimilar vector status (*Cx. quinquefasciatus* and *Cx. sitiens*). This accuracy of MALDI-TOF MS in mosquito species identification can also be exploited to circumvent limitations of morphological method in identifying damaged specimens. It also represents an interesting complementary method in mosquito identification, all the more that experts in systematics have decreased resulting from lack of expertise transmission.

In this study, the optimal threshold for mosquito identification which maximized both MALDI-TOF MS sensitivity and specificity using ROC analysis was determined. The higher threshold value was found for *Cx. quinquefasciatus* (i.e. 1.79). The optimal threshold value remained comparable to the results for each species when half of the database was included in the analysis (1.63 *vs* 1.70). High positive and negative predictive values at these thresholds show the ability of the MALDI-TOF MS to correctly differentiate mosquito species (Table 2). Based on the results and for optimal identification, the threshold value was rounded to 1.8. The same threshold value as previously stated was therefore attained [17, 19, 24]. At this threshold value, 92% of mosquitoes were correctly identified.

All these findings suggest that MALDI-TOF MS is reliable for mosquito identification. In addition, the comparison of cost and workload requirement between MALDI-TOF MS analysis and molecular identification during experimentation confirmed other authors’ findings [22]. In terms of required expenses, MALDI-TOF MS is much cheaper than the molecular technique (about 1 Euro *vs* 20 Euro per sample in these experiments). Moreover, mosquito species identification by MALDI-TOF MS can be obtained after an eight-hour procedure (starting from protein extraction to obtaining of results), which is significantly shorter than the time needed to perform molecular identification (about one week, starting from DNA extraction to sequence analysis).

This study may suffer from bias and limitations. The first possible limitation of the study is the low number of MSPs for *An. bancroftii* due to their insufficient number in the reference database. In spite of that, high identification scores for six of the seven *An. bancroftii* used for the blind testing were achieved. In the future, other samples should be added to the database to have at least five MSPs.

Secondly, only legs were used for protein extraction during this study. These are more breakable during trapping or storage. As observed during analysis, the use of less than four legs for protein extraction could compromise MALDI-TOF identification. Another study has suggested the use of the thorax, which is not prone to degradation during sampling [24]. The creation of another database using mosquito thoraxes for these species should be undertaken in the future to strengthen MALDI-TOF identification, in case few or no legs are available.

Thirdly, unlike other studies during which several spots per sample was realized with consideration of the highest LSV, one single spot for each sample was deposited in order to assess each parameter individually (trapping duration, preservation methods and duration, number of MSPs required for MALDI-TOF database creation and threshold value determination) without score improvement caused by depositing multiple spots. Consequently, the LSVs obtained are not optimized. Indeed, supplementary experiments showed that the LSVs improved when three spots of each sample were deposited onto the target plate with consideration of the highest scoring spectrum within the triplets (Additional file 6: Figure S6). However, with one single spot, 92% of these mosquitoes identified showed an identification LSV that was higher than the threshold (1.8) during the blind test analysis. In the future, at least two spots per sample should be deposited, especially when using MALDI-TOF MS to identify newly introduced species.

Fourthly and finally, the optimal threshold was determined using the current MALDI-TOF database. The determination of this threshold score could depend on the richness of species diversity in the database. Adding more species in this database could potentially increase or decrease this threshold value. This value should therefore be revised when more species belonging to the same group become included in analyses.

## Conclusions

In conclusion, MALDI-TOF MS is a highly sensitive and highly specific method for mosquito identification in New Caledonia. This emerging identification method is a promising tool in public health for mosquito vector surveillance, allowing both accurate identification of mosquito species vectors in a region and rapid detection of introduced vector species. Other vector mosquito species recorded in the Pacific region including the other *Aedes* members of the *scutellaris* group are not present in this MALDI-TOF MS database. The inclusion of these species in the database is needed for MALDI-TOF to be used as an effective entomological surveillance and risk assessment tool in the Pacific region. Thus, this MALDI-TOF MS database will be able to provide a rapid response to vectors identification/introduction and help public health responses.

## Supplementary information

**Additional file 1: Figure S1.** MALDI-TOF MS spectra of fresh mosquitoes and those harvested from BG-sentinel at each same point.

**Additional file 2: Figure S2.** MSP dendrogram of all the mosquitoes included in the New Caledonian MALDI-TOF database (*n* = 67).

**Additional file 3: Figure S3.** ROC to determine the threshold value which maximizes the sensitivity and specificity of MALDI-TOF MS for mosquito species identification.

**Additional file 4: Figure S4.** Impact of preservation duration on *Ae. aegypti* identification scores.

**Additional file 5: Figure S5.** Comparison of LSVs of *Ae. aegypti* collected in 2015 and preserved for four years at − 80 °C (*n* = 20) and LSVs of *Ae. aegypti* collected in 2018 and analyzed no later than five months following sampling (*n* = 30).

**Additional file 6: Figure S6.** Comparison of log-score values according to number of spots per sample.

## Data Availability

Data supporting the conclusions of this article are included within the article and its additional files. Raw data are available from the corresponding authors upon reasonable request. Sequences have been deposited in the GenBank database under the accession numbers MN733743-MN733814.

## Abbreviations

CDC: Center for Diseases, Control and Prevention
*cox*1: cytochrome *c* oxidase subunit 1
DNA: deoxyribonucleic acid
ITS2: internal transcribed spacer 2
LSV: log-score value
MALDI-TOF MS: matrix-assisted laser desorption/ionization time-of-flight mass spectrometry
MSP: main spectrum pattern
PCR: polymerase chain reaction
ROC: receiver operating characteristics

## References

[1] WHO. Dengue and severe dengue. Geneva: World Health Organization; 2019. https://www.who.int/respiratory/asthma/en/.

[2] Cao-Lormeau VM, Musso D (2014). Emerging arboviruses in the Pacific. Lancet..

[3] Espinal MA, Andrus JK, Jauregui B, Waterman SH, Morens DM, Santos JI (2019). Emerging and reemerging *Aedes*-transmitted arbovirus infections in the region of the Americas: implications for health policy. Am J Public Health..

[4] Inizan C, Tarantola A, O’Connor O, Mangeas M, Pocquet N, Forfait C (2019). Dengue in New Caledonia: knowledge and gaps. Trop Med Infect Dis..

[5] Dupont-Rouzeyrol M, O’Connor O, Calvez E, Daures M, John M, Grangeon JP (2015). Co-infection with Zika and dengue viruses in 2 patients, New Caledonia, 2014. Emerg Infect Dis..

[6] Watson TM, Kay BH (1998). Vector competence of *Aedes notoscriptus* (Diptera: Culicidae) for Ross River virus in Queensland. Australia. J Med Entomol..

[7] Turell MJ, Kay BH (1998). Susceptibility of selected strains of Australian mosquitoes (Diptera: Culicidae) to Rift Valley fever virus. J Med Entomol..

[8] Richards SL, Anderson SL, Lord CC (2014). Vector competence of *Culex pipiens quinquefasciatus* (Diptera: Culicidae) for West Nile virus isolates from Florida. Trop Med Int Health..

[9] Van Den Hurk AF, Nisbet DJ, Hall RA, Kay BH, Mackenzie JS, Ritchie SA (2003). Vector competence of Australian mosquitoes (Diptera: Culicidae) for Japanese encephalitis virus. J Med Entomol..

[10] Vythilingam I, Tan SB, Krishnasamy M (2002). Short communication: susceptibility of *Culex sitiens* to Japanese encephalitis virus in peninsular Malaysia. Trop Med Int Health..

[11] Belkin JN (1962). The mosquitoes of the south pacific (Diptera, Culicidae).

[12] Guillaumot L (2005). Arboviruses and their vectors in the Pacific—status reports. Pac Health Dialog..

[13] Guillaumot L, Ofanoa R, Swillen L, Singh N, Bossin HC, Schaffner F (2012). Distribution of *Aedes albopictus* (Diptera, Culicidae) in southwestern Pacific countries, with a first report from the Kingdom of Tonga. Parasit Vectors..

[14] Rapport d’activité -Institut Pasteur de Nouvelle-Calédonie, Nouvelle-Calédonie; 2016. http://www.institutpasteur.nc/wp-content/uploads/2016/04/Rapport2016Final.pdf. Accessed 5 July 2020.

[15] Pol M, Kilama S, Duperier S, Soupé-Gilbert ME, Calvez E, Pocquet N (2018). Introduction of the *Anopheles bancroftii* mosquito, a malaria vector, into New Caledonia. Emerg Infect Dis..

[16] WHO. Global Vector Control Response 2017–2030. Geneva: World Health Organization; 2017. https://apps.who.int/iris/handle/10665/259205.

[17] Yssouf A, Almeras L, Raoult D, Parola P (2016). Emerging tools for identification of arthropod vectors. Future Microbiol..

[18] Kent RJ, Deus S, Williams M, Savage HM (2010). Development of a multiplexed polymerase chain reaction-restriction fragment length polymorphism (PCR-RFLP) assay to identify common members of the subgenera *Culex* (*Culex*) and *Culex* (*Phenacomyia*) in Guatemala. Am J Trop Med Hyg.

[19] Yssouf A, Socolovschi C, Flaudrops C, Ndiath MO, Sougoufara S, Dehecq JS (2013). Matrix-assisted laser desorption ionization—time of flight mass spectrometry: an emerging tool for the rapid identification of mosquito vectors. PLoS ONE..

[20] Müller P, Pflüger V, Wittwer M, Ziegler D, Chandre F, Simard F (2013). Identification of cryptic *Anopheles* mosquito species by molecular protein profiling. PLoS ONE..

[21] Bizzini A, Durussel C, Bille J, Greub G, Prod’hom G (2010). Performance of matrix-assisted laser desorption ionization-time of flight mass spectrometry for identification of bacterial strains routinely isolated in a clinical microbiology laboratory. J Clin Microbiol..

[22] Carbonnelle E, Mesquita C, Bille E, Day N, Dauphin B, Beretti JL (2011). MALDI-TOF mass spectrometry tools for bacterial identification in clinical microbiology laboratory. Clin Biochem..

[23] Halada P, Hlavačková K, Risueño J, Berriatua E, Volf P, Dvorak V (2018). Effect of trapping method on species identification of phlebotomine sandflies by MALDI-TOF MS protein. Med Vet Entomol..

[24] Vega-Rúa A, Pagès N, Fontaine A, Nuccio C, Hery L, Goindin D (2018). Improvement of mosquito identification by MALDI-TOF MS biotyping using protein signatures from two body parts. Parasit Vectors..

[25] Nebbak A, El Hamzaoui B, Berenger JM, Bitam I, Raoult D, Almeras L (2017). Comparative analysis of storage conditions and homogenization methods for tick and flea species for identification by MALDI-TOF MS. Med Vet Entomol..

[26] Niare S, Berenger JM, Dieme C, Doumbo O, Raoult D, Parola P (2016). Identification of blood meal sources in the main African malaria mosquito vector by MALDI-TOF MS. Malar J..

[27] Belkin JN (1962). The mosquitoes of the south pacific (Part B).

[28] Rueda LM (2004). Pictorial keys for the identification of mosquitoes (Diptera: Culicidae) associated with dengue virus transmission. Zootaxa..

[29] Lee DJ, Debenham ML (1987). The Culicidae of the Australian region.

[30] Folmer O, Black M, Hoeh W, Lutz R, Vrijenhoek R (1994). DNA primers for amplification of mitochondrial cytochrome *c* oxidase subunit I from diverse metazoan invertebrates. Mol Mar Biol Biotechnol..

[31] Beebe NW, Maung J, Van Den Hurk AF, Ellis JT, Cooper RD (2001). Ribosomal DNA spacer genotypes of the *Anopheles bancroftii* group (Diptera: Culicidae) from Australia and Papua New Guinea. Insect Mol Biol..

[32] Raharimalala FN, Andrianinarivomanana TM, Rakotondrasoa A, Collard JM, Boyer S (2017). Usefulness and accuracy of MALDI-TOF mass spectrometry as a supplementary tool to identify mosquito vector species and to invest in development of international database. Med Vet Entomol..

[33] Feltens R, Gorner R, Kalkhof S, Groger-Arndt H, von Bergen M (2010). Discrimination of different species from the genus *Drosophila* by intact protein profiling using matrix-assisted laser desorption ionization mass spectrometry. BMC Evol Biol..

[34] Diarra AZ, Almeras L, Laroche M, Berenger JM, Koné AK, Bocoum Z (2017). Molecular and MALDI-TOF identification of ticks and tick-associated bacteria in Mali. PLoS Negl Trop Dis..

[35] Zurita A, Djeghar R, Callejón R, Cutillas C, Parola P, Laroche M (2019). Matrix-assisted laser desorption/ionization time-of-flight mass spectrometry as a useful tool for the rapid identification of wild flea vectors preserved in alcohol. Med Vet Entomol..

[36] Yu D, Klein S, Reindl D (2001). An evaluation of silica gel for humidity control in display cases. WAAC Newslett.

[37] Calvez E, Guillaumot L, Millet L, Marie J, Bossin H, Rama V (2016). Genetic diversity and phylogeny of *Aedes aegypti*, the main arbovirus vector in the Pacific. PLoS Negl Trop Dis..

[38] Sauer S, Freiwald A, Maier T, Kube M, Reinhardt R, Kostrzewa M (2008). Classification and identification of bacteria by mass spectrometry and computational analysis. PLoS ONE..

