# Supplementary material for: MALDI-TOF MS: optimization for future uses in entomological surveillance and identification of mosquitoes from New Caledonia
Source: Parasit Vectors. 2020 Jul 20;13:359. doi: 10.1186/s13071-020-04234-8 (PMC7372833; doi:10.1186/s13071-020-04234-8)
Supplement: Supplementary file 1 — Additional file 1: Figure S1. MALDI-TOF MS spectra of fresh mosquitoes and those harvested from BG-sentinel at each same point. [file 13071_2020_4234_MOESM1_ESM.pdf]

**Additional file 1: Figure S1.** MALDI-TOF MS spectra of fresh mosquitoes and those harvested from BG-sentinel at each same point. Spectra from three specimens per point are shown on each graphic: fresh mosquitoes (**a**); mosquitoes harvested at 24 h (**b**); mosquitoes harvested at 48 h (**c**); mosquitoes harvested at 72 h (**d**); mosquitoes harvested at 96 h (**e**); and mosquitoes harvested at 168 h (**f**). X-axis corresponds to mass to charge ration (M/z). Y-axis corresponds to relative intensity of signal. Colours illustrate each individual for each group. *Abbreviation*: AU, arbitrary units.

# A

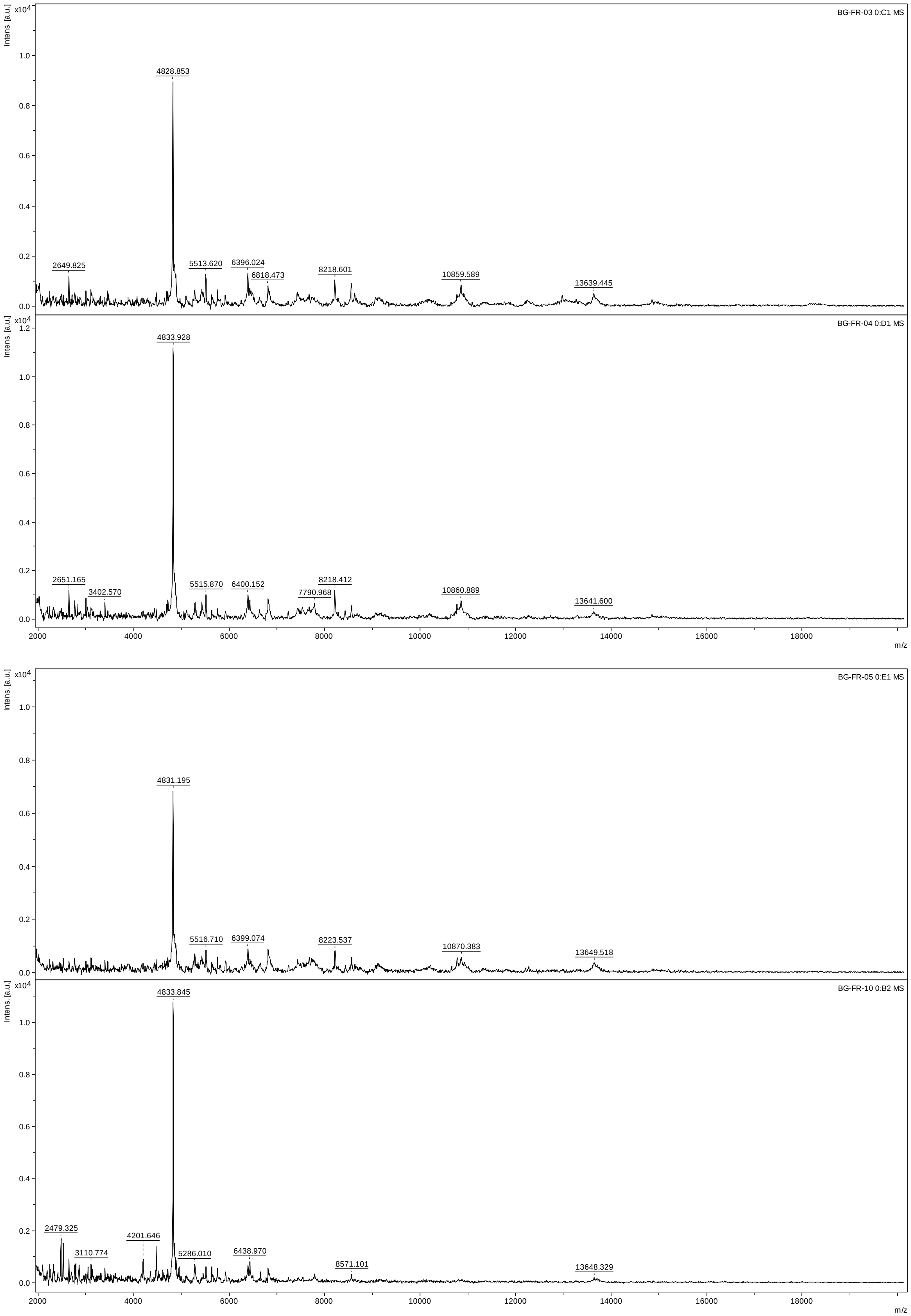

x10^4
Intens. [a.u.]
BG-FR-03 0:C1 MS
1.0
0.8
0.6
0.4
0.2
0.0
4828.853
2649.825
5513.620
6396.024
6818.473
8218.601
10859.589
13639.445
x10^4
Intens. [a.u.]
BG-FR-04 0:D1 MS
1.2
1.0
0.8
0.6
0.4
0.2
0.0
4833.928
2651.165
3402.570
5515.870
6400.152
7790.968
8218.412
10860.889
13641.600
2000
4000
6000
8000
10000
12000
14000
16000
18000
m/z
x10^4
Intens. [a.u.]
BG-FR-05 0:E1 MS
1.0
0.8
0.6
0.4
0.2
0.0
4831.195
5516.710
6399.074
8223.537
10870.383
13649.518
x10^4
Intens. [a.u.]
BG-FR-10 0:B2 MS
1.0
0.8
0.6
0.4
0.2
0.0
4833.845
2479.325
3110.774
4201.646
5286.010
6438.970
8571.101
13648.329
2000
4000
6000
8000
10000
12000
14000
16000
18000
m/z

**B**

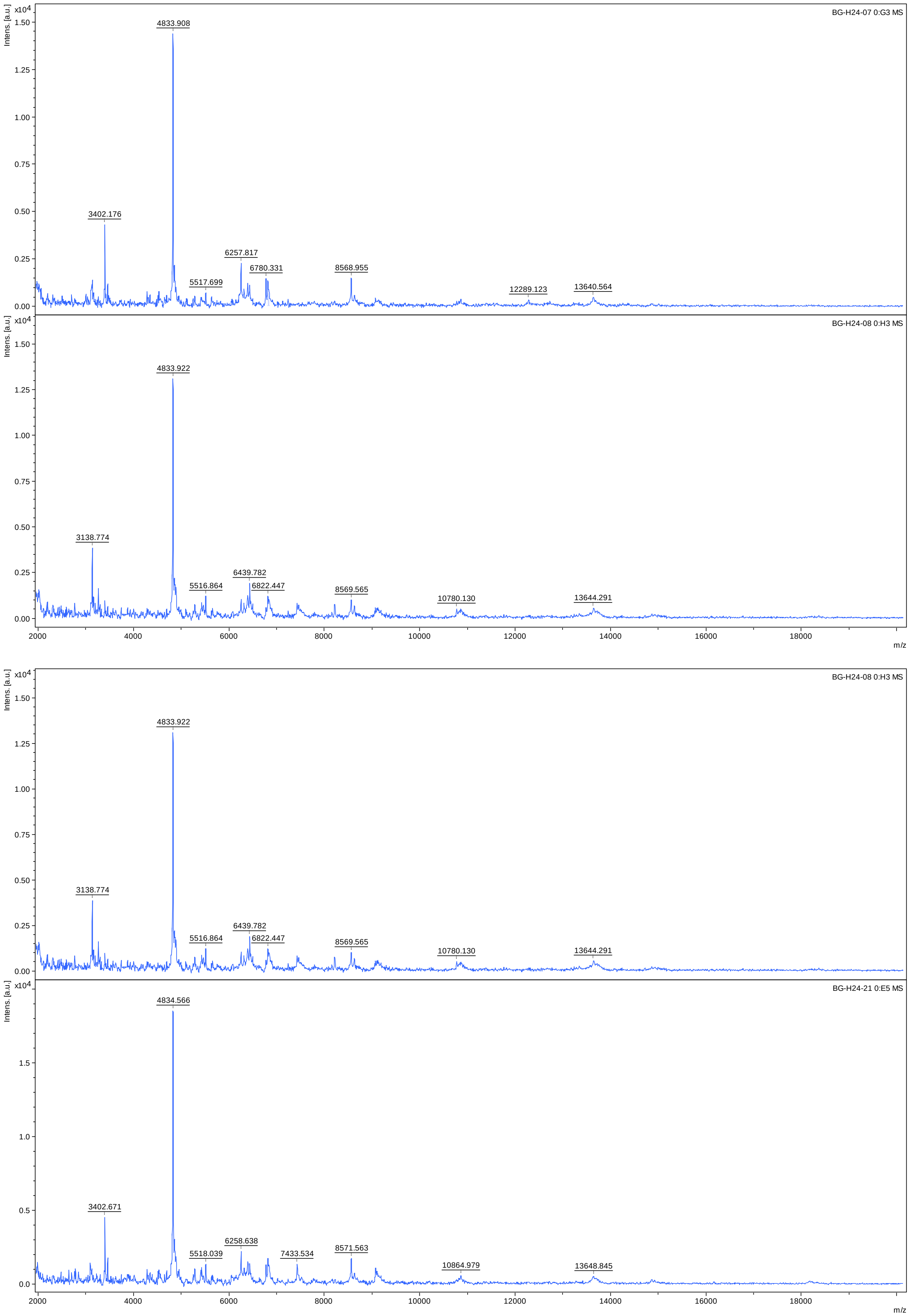
BG-H24-07 0:G3 MS
4833.908
3402.176
6257.817
6780.331
5517.699
8568.955
12289.123
13640.564
BG-H24-08 0:H3 MS
4833.922
3138.774
6439.782
5516.864
6822.447
8569.565
10780.130
13644.291
BG-H24-08 0:H3 MS
4833.922
3138.774
6439.782
5516.864
6822.447
8569.565
10780.130
13644.291
BG-H24-21 0:E5 MS
4834.566
3402.671
6258.638
5518.039
7433.534
8571.563
10864.979
13648.845
Intens. [a.u.]
x10^4
m/z

**C**

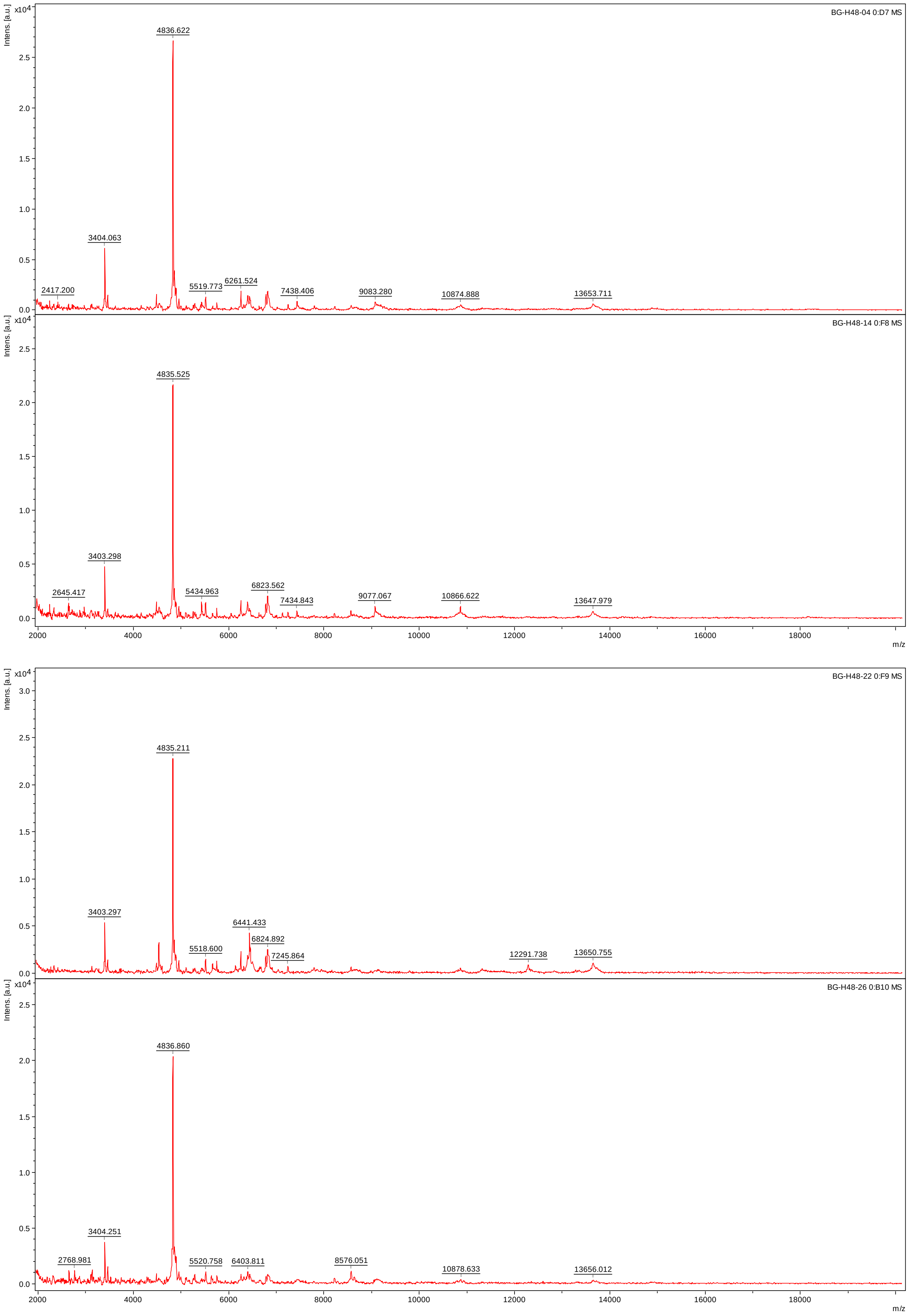

BG-H48-04 0:D7 MS
4836.622
3404.063
2417.200
5519.773
6261.524
7438.406
9083.280
10874.888
13653.711
BG-H48-14 0:F8 MS
4835.525
3403.298
2645.417
5434.963
6823.562
7434.843
9077.067
10866.622
13647.979
BG-H48-22 0:F9 MS
4835.211
3403.297
6441.433
6824.892
5518.600
7245.864
12291.738
13650.755
BG-H48-26 0:B10 MS
4836.860
3404.251
2768.981
5520.758
6403.811
8576.051
10878.633
13656.012
Intens. [a.u.]
x10^4
m/z

**D**

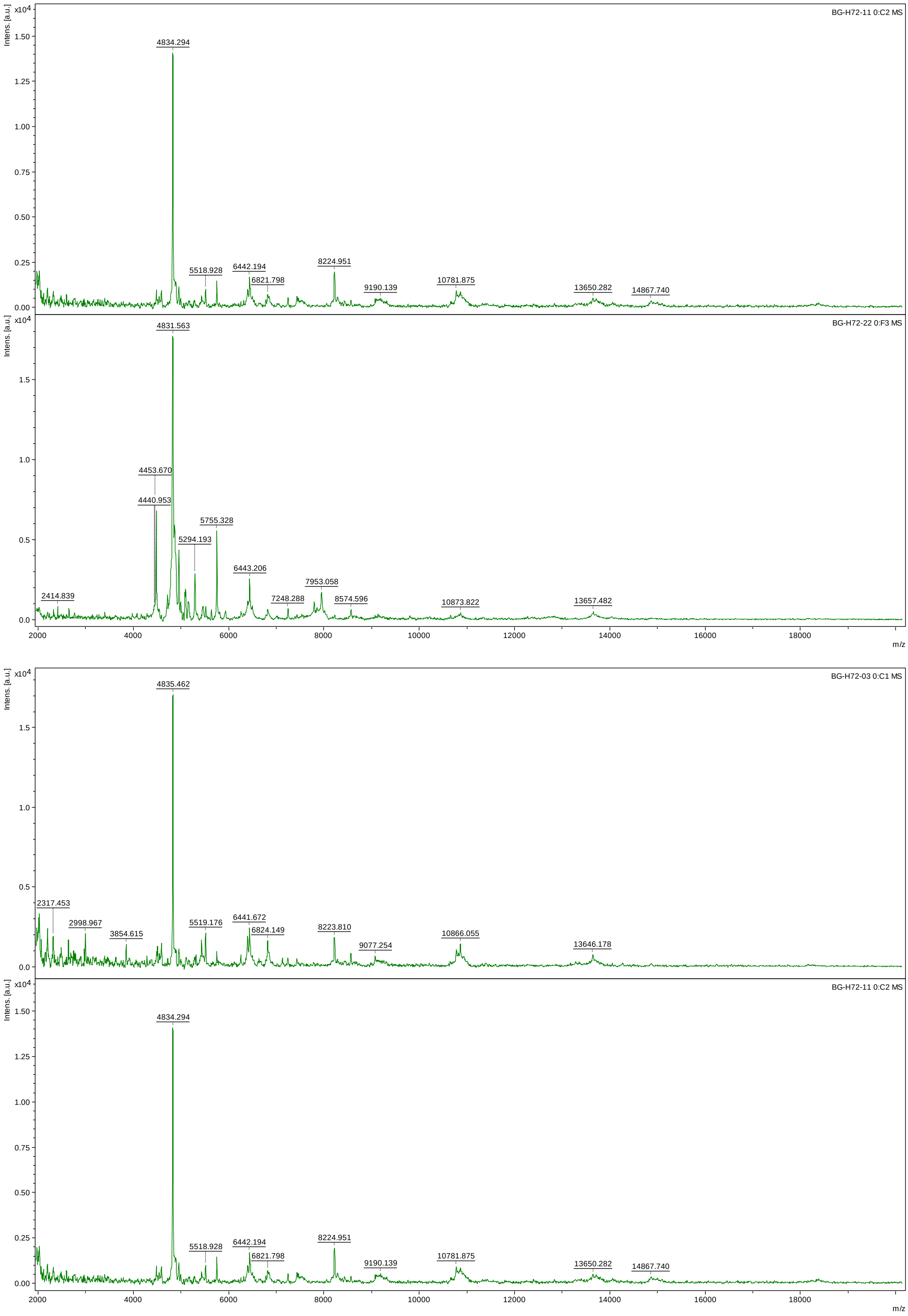
BG-H72-11 0:C2 MS
Intens. [a.u.]
x10^4
4834.294
5518.928
6442.194
6821.798
8224.951
9190.139
10781.875
13650.282
14867.740
BG-H72-22 0:F3 MS
4831.563
4453.670
4440.953
5755.328
5294.193
6443.206
7953.058
7248.288
8574.596
2414.839
10873.822
13657.482
m/z
BG-H72-03 0:C1 MS
4835.462
2317.453
2998.967
3854.615
5519.176
6441.672
6824.149
8223.810
9077.254
10866.055
13646.178
BG-H72-11 0:C2 MS
4834.294
5518.928
6442.194
6821.798
8224.951
9190.139
10781.875
13650.282
14867.740
m/z

**E**

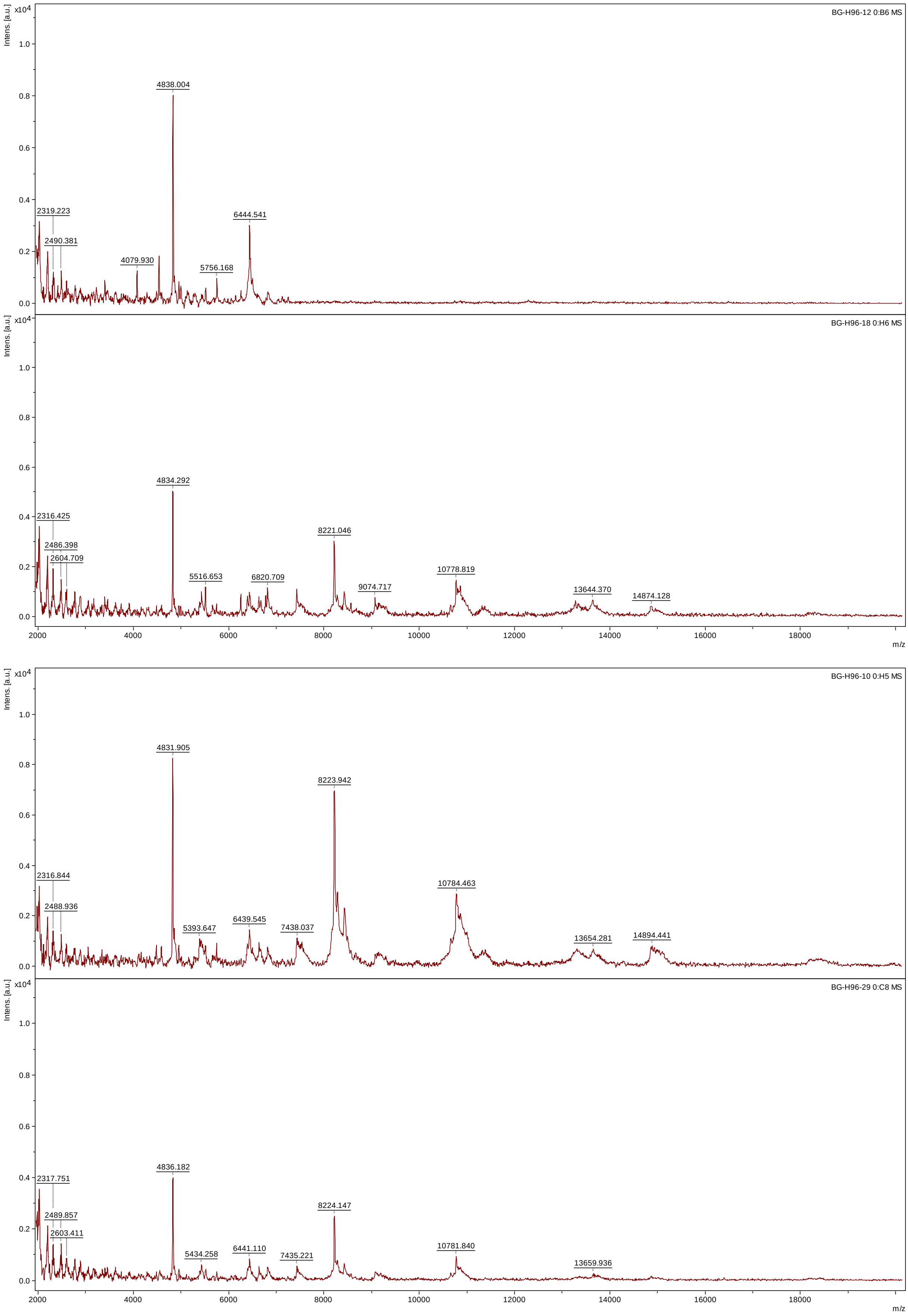

BG-H96-12 0:B6 MS
Intens. [a.u.]
x10^4
2319.223
2490.381
4079.930
4838.004
5756.168
6444.541
BG-H96-18 0:H6 MS
2316.425
2486.398
2604.709
4834.292
5516.653
6820.709
8221.046
9074.717
10778.819
13644.370
14874.128
m/z
BG-H96-10 0:H5 MS
2316.844
2488.936
4831.905
5393.647
6439.545
7438.037
8223.942
10784.463
13654.281
14894.441
BG-H96-29 0:C8 MS
2317.751
2489.857
2603.411
4836.182
5434.258
6441.110
7435.221
8224.147
10781.840
13659.936

**F**

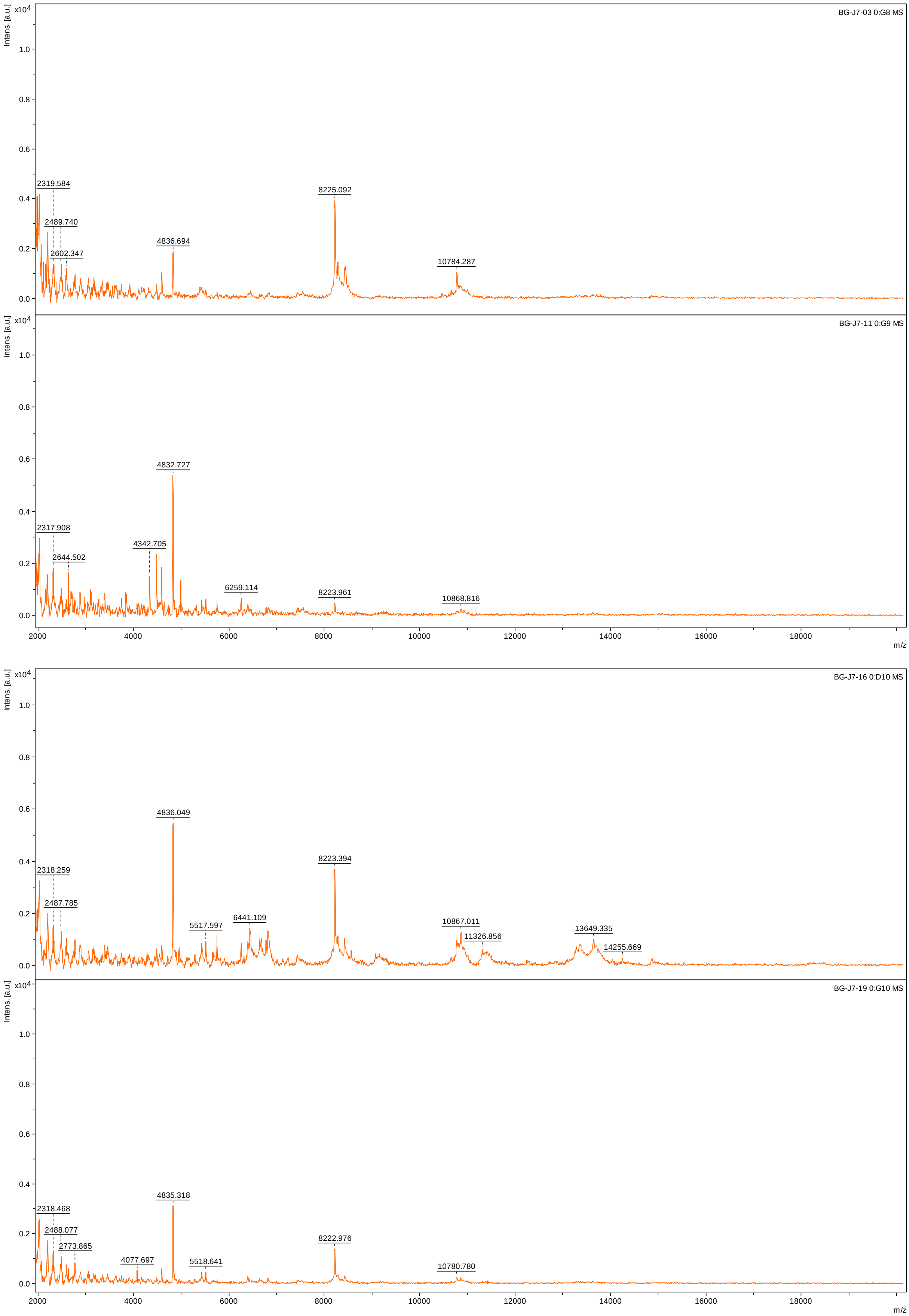
x10^4
Intens. [a.u.]
BG-J7-03 0:G8 MS
1.0
0.8
0.6
0.4
0.2
0.0
2319.584
2489.740
2602.347
4836.694
8225.092
10784.287
BG-J7-11 0:G9 MS
2317.908
2644.502
4342.705
4832.727
6259.114
8223.961
10868.816
2000
4000
6000
8000
10000
12000
14000
16000
18000
m/z
BG-J7-16 0:D10 MS
2318.259
2487.785
4836.049
5517.597
6441.109
8223.394
10867.011
11326.856
13649.335
14255.669
BG-J7-19 0:G10 MS
2318.468
2488.077
2773.865
4077.697
4835.318
5518.641
8222.976
10780.780
