# Supplementary material for: MALDI-TOF MS: optimization for future uses in entomological surveillance and identification of mosquitoes from New Caledonia
Source: Parasit Vectors. 2020 Jul 20;13:359. doi: 10.1186/s13071-020-04234-8 (PMC7372833; doi:10.1186/s13071-020-04234-8)

Additional file 2: Figure S2. MSP dendrogram of all the mosquitoes included in the New Caledonian MALDI-TOF database (*n* = 67). The dendrogram was created using Biotyper v3.0 software. Distance units correspond to the relative similarity of MS spectra. Distance levels under 500 correspond to a reliable classification to species level.

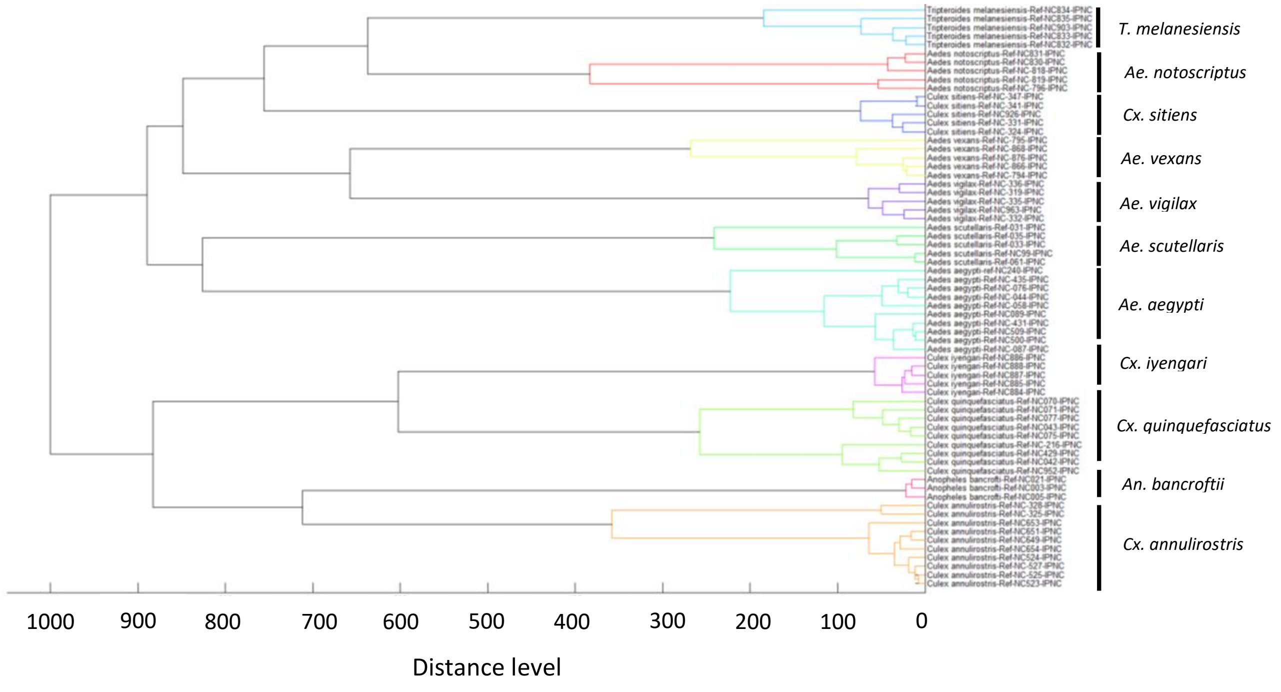

Supplement: Supplementary file 2 — Additional file 2: Figure S2. MSP dendrogram of all the mosquitoes included in the New Caledonian MALDI-TOF database (n = 67). [file 13071_2020_4234_MOESM2_ESM.pdf]
