# Supplementary material for: MALDI-TOF MS: optimization for future uses in entomological surveillance and identification of mosquitoes from New Caledonia
Source: Parasit Vectors. 2020 Jul 20;13:359. doi: 10.1186/s13071-020-04234-8 (PMC7372833; doi:10.1186/s13071-020-04234-8)

Additional file 3: Figure S3. ROC to determine the threshold value which maximizes the sensitivity and specificity of MALDI-TOF MS for mosquito species identification. These results were obtained when a random selection of six species (*An. bancroftii*, *Ae. scutellaris*, *Ae. aegypti*, *Ae. vigilax*, *Cx. quinquefasciatus* and *Cx. sitiens*) (**a**) and five other species (*Ae. notoscriptus*, *Ae. vexans*, *Cx. iyengari*, *Cx. annulirostris* and *T. melanesiensis* (**b**) were included in the database. X-axis corresponds to threshold value. Y-axis corresponds to sensitivity and specificity of the MALDI-TOF technique.

A

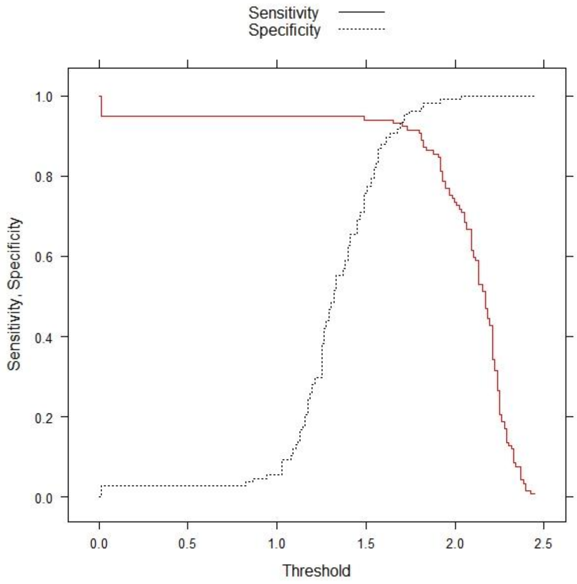

B

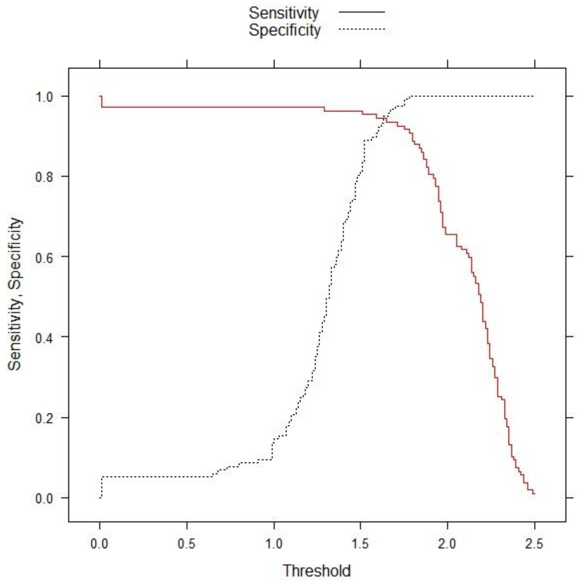

Supplement: Supplementary file 3 — Additional file 3: Figure S3. ROC to determine the threshold value which maximizes the sensitivity and specificity of MALDI-TOF MS for mosquito species identification. [file 13071_2020_4234_MOESM3_ESM.pdf]
