# Supplementary material for: MALDI-TOF MS: optimization for future uses in entomological surveillance and identification of mosquitoes from New Caledonia
Source: Parasit Vectors. 2020 Jul 20;13:359. doi: 10.1186/s13071-020-04234-8 (PMC7372833; doi:10.1186/s13071-020-04234-8)
Supplement: Supplementary file 4 — Additional file 4: Figure S4. Impact of preservation duration on Ae. aegypti identification scores. [file 13071_2020_4234_MOESM4_ESM.pdf]

Additional file 4: Figure S4. Impact of preservation duration on *Ae. aegypti* identification scores. Comparison of mosquitoes preserved in 70% ethanol (*n* = 18 to 30 per point) with one MSP created from a specimen preserved in ethanol for one day (**a**). Comparison of frozen mosquitoes (*n* = 30 per point) with one MSP created from a specimen preserved at -20 °C for one day (**b**). Comparison of mosquito preserved in silica gel and cotton at room temperature (*n* = 20 to 30 per point) with one MSP created from a specimen preserved silica gel and cotton for one day (**c**). X-axis corresponds to preservation duration. Y-axis corresponds to log-score values (LSVs) obtained. Wilcoxon test, $^{**}P < 0.01$, $^{***}P < 0.001$. *Abbreviation*: NS, not significant.

A

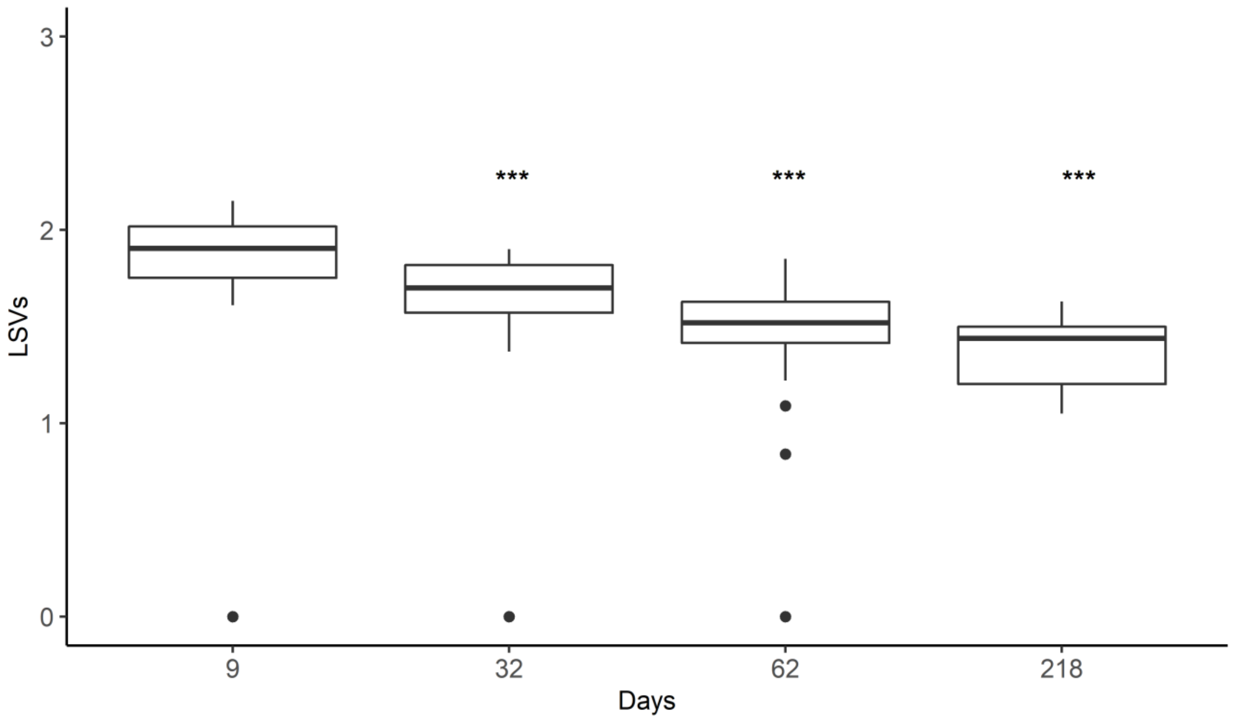

B

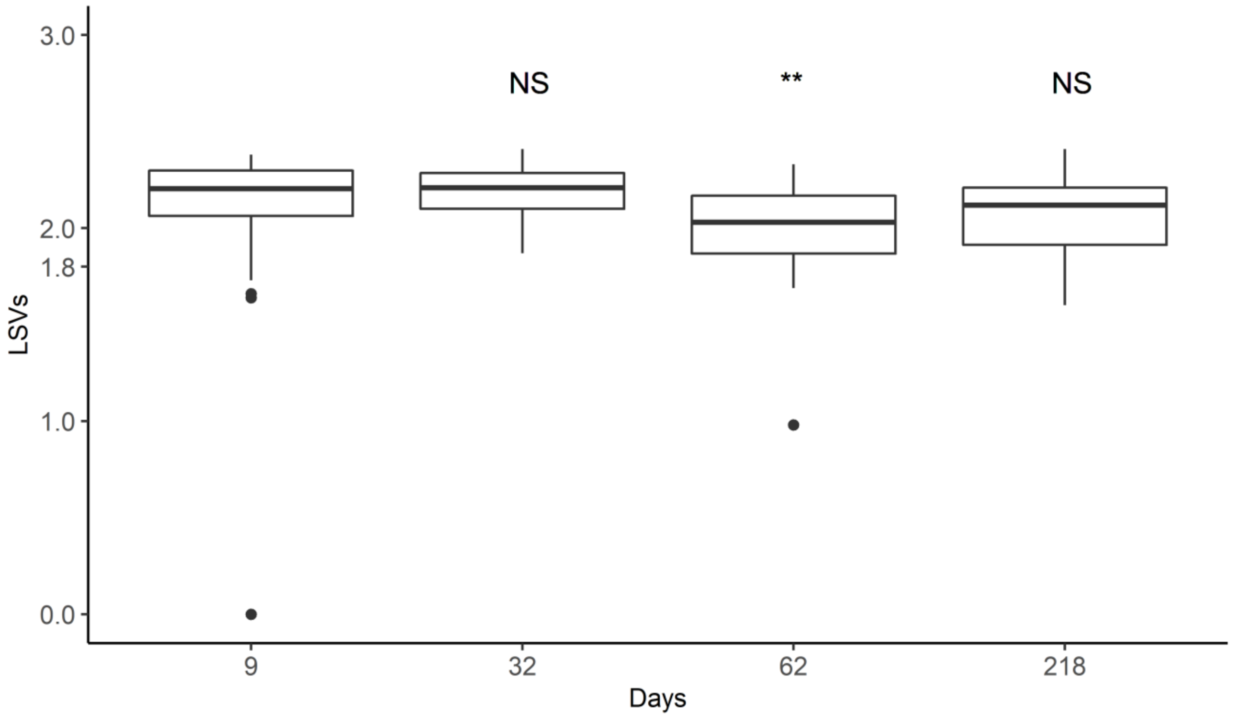
NS
**
NS
3.0
2.0
1.8
1.0
0.0
LSVs
9
32
62
218
Days

C

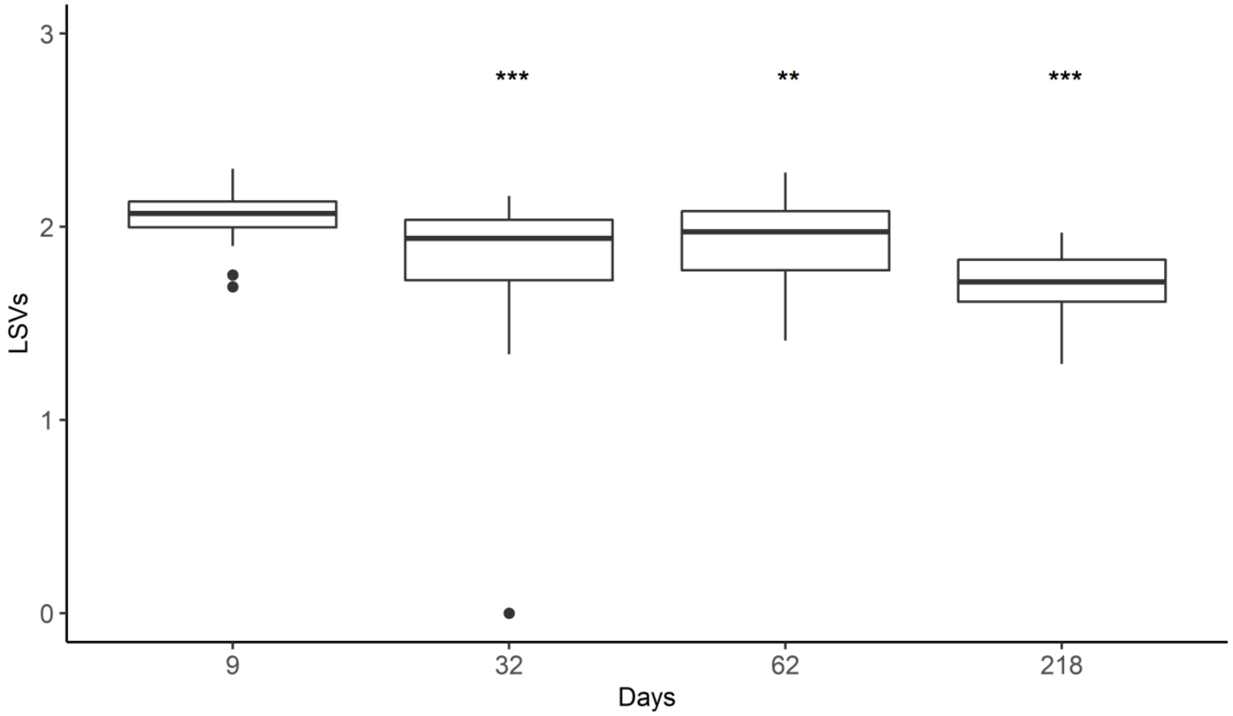
***
**
***
3
2
1
0
LSVs
9
32
62
218
Days
