# Supplementary material for: MALDI-TOF MS: optimization for future uses in entomological surveillance and identification of mosquitoes from New Caledonia
Source: Parasit Vectors. 2020 Jul 20;13:359. doi: 10.1186/s13071-020-04234-8 (PMC7372833; doi:10.1186/s13071-020-04234-8)

Additional file 5: Figure S5. Comparison of LSVs of *Ae. aegypti* collected in 2015 and preserved for four years at -80 °C (n = 20) and LSVs of *Ae. aegypti* collected in 2018 and analyzed no later than five months following sampling (n = 30). All mosquitoes collected in 2018 were preserved at -20 °C or at -80 °C if preservation exceeded three weeks before MALDI-TOF MS analysis. Spectra obtained with all these mosquitoes were compared to MSPs created from fresh field-collected specimens in the database. Correct identification was achieved with high LSVs. On the graph, colors illustrate MALDI-TOF MS identification, the red dashed line marks the threshold value of 1.8 and the green lines illustrate LSV median for each group.

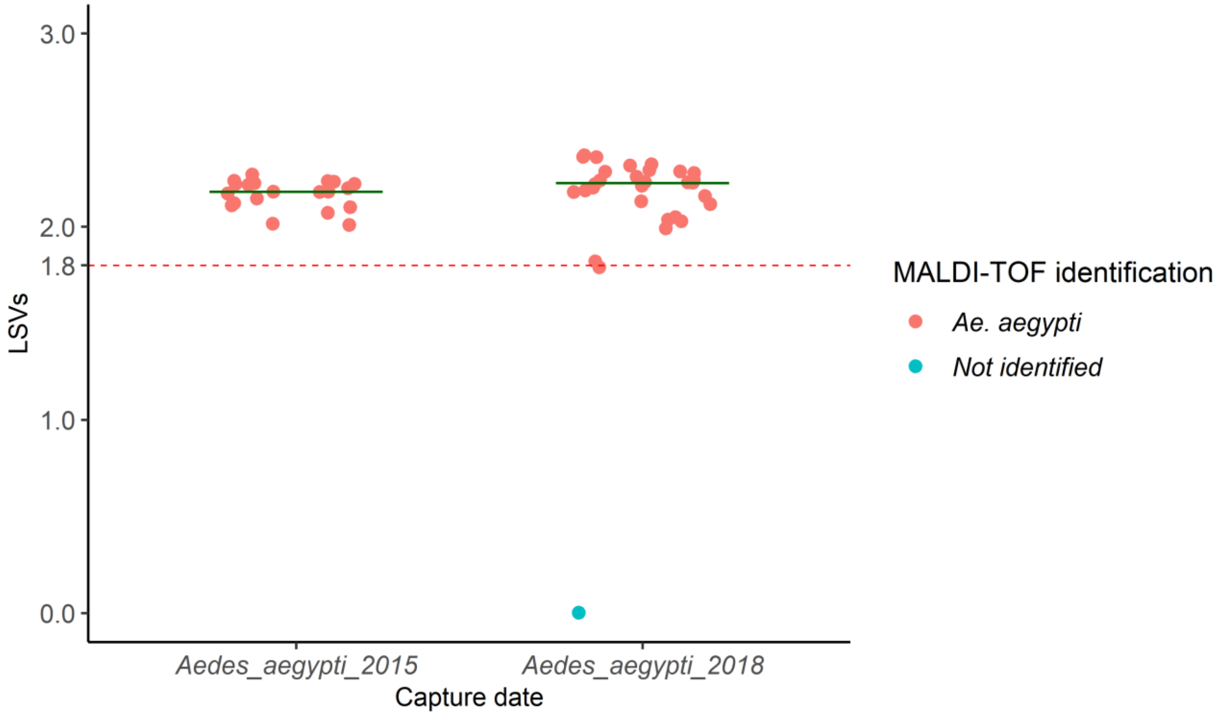

Supplement: Supplementary file 5 — Additional file 5: Figure S5. Comparison of LSVs of Ae. aegypti collected in 2015 and preserved for four years at − 80 °C (n = 20) and LSVs of Ae. aegypti collected in 2018 and analyzed no later than five months following sampling (n = 30). [file 13071_2020_4234_MOESM5_ESM.pdf]
