# Supplementary material for: MALDI-TOF MS: optimization for future uses in entomological surveillance and identification of mosquitoes from New Caledonia
Source: Parasit Vectors. 2020 Jul 20;13:359. doi: 10.1186/s13071-020-04234-8 (PMC7372833; doi:10.1186/s13071-020-04234-8)

Additional file 6: Figure S6. Comparison of log-score values according to number of spots per sample. Analysis was performed using *Ae. aegypti* from the field (n = 29) which were compared with MSPs in the database. The red dashed line marks the threshold value of 1.8. Wilcoxon test, **P < 0.01. *Abbreviations*: NS, not significant; LSVs, log-score values.

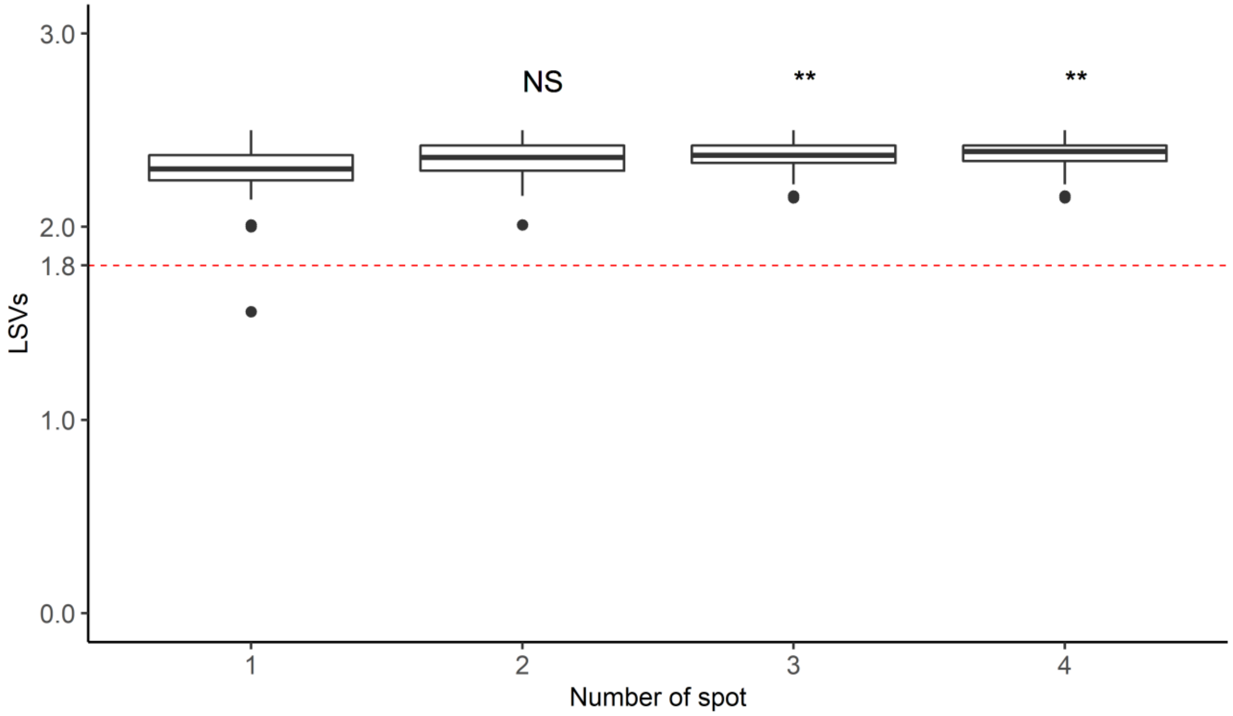

Supplement: Supplementary file 6 — Additional file 6: Figure S6. Comparison of log-score values according to number of spots per sample. [file 13071_2020_4234_MOESM6_ESM.pdf]
